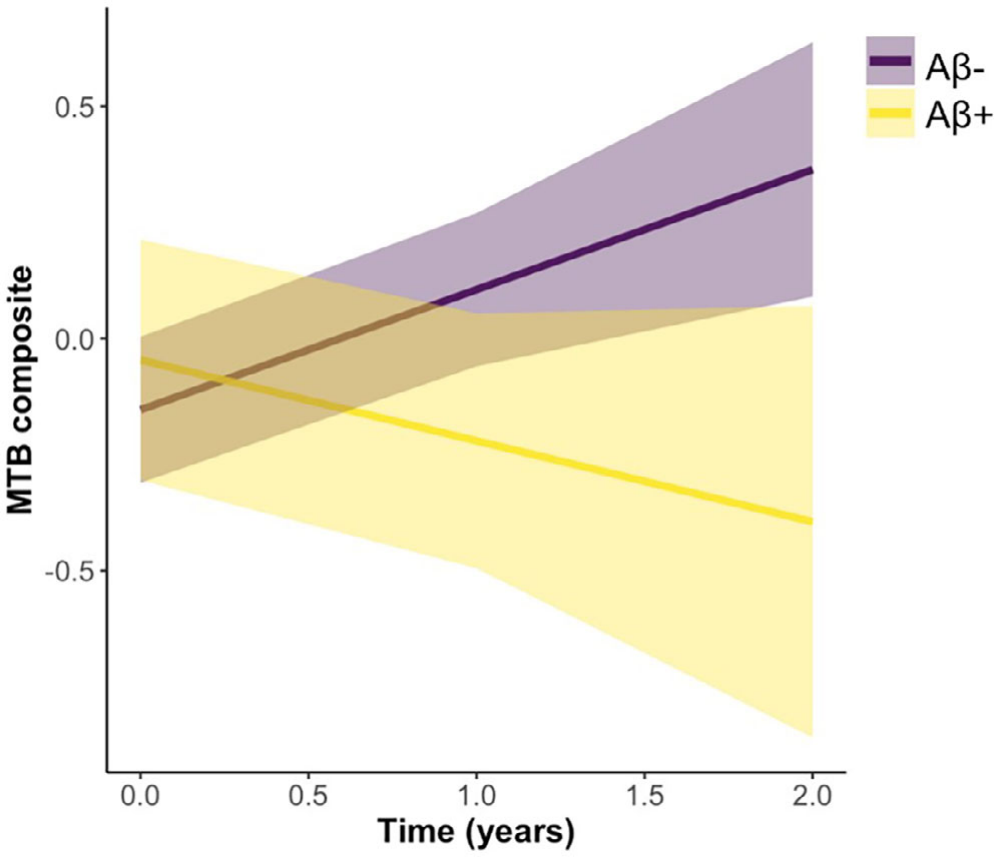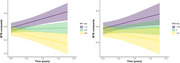# The remote Mobile Toolbox for capturing cognitive change in preclinical Alzheimer’s disease

**DOI:** 10.1002/alz70861_108826

**Published:** 2025-12-23

**Authors:** Roos J Jutten, Jessa Burling, Elliott Slade, Jackson C Thompson, Michael J. Properzi, Jessie Fanglu Fu, Gad A. Marshall, Rebecca E. Amariglio, Keith A. Johnson, Julie C Price, Reisa A. Sperling, Kathryn V Papp, Dorene M. Rentz

**Affiliations:** ^1^ Alzheimer Center Amsterdam, Neurology, Vrije Universiteit Amsterdam, Amsterdam UMC location VUmc, Amsterdam Netherlands; ^2^ Massachusetts General Hospital, Harvard Medical School, Boston, MA USA; ^3^ Brigham and Women's Hospital, Harvard Medical School, Boston, MA USA

## Abstract

**Background:**

Remote, smartphone‐based cognitive assessments such as the Mobile Toolbox (MTB) may facilitate the accessibility and scalability of Alzheimer’s disease (AD) research and clinical trials. We previously showed that a composite of MTB memory and processing speed tests provides a feasible approach to measure cognitive processes that are relevant in preclinical AD. Here, we examined whether this MTB‐composite is sensitive to amyloid‐ and tau‐related cognitive change over time.

**Method:**

The MTB was deployed longitudinally in N=100 cognitively unimpaired (CU) older adults from four affiliated observational cohort‐studies. All participants self‐completed the MTB remotely on their personal mobile device. Our MTB‐composite of interest includes two measures of episodic memory (Arranging Pictures and Faces & Names) and two measures of attention and processing speed (Sequences and Number‐Symbol Match). Amyloid‐β positron emission tomography (PET) and tau PET were available within ‐1.56±1.97 years of the first MTB assessment. Global amyloid‐β burden was dichotomized into negative (Aβ‐) versus positive (Aβ+) using a distribution volume ratio cut‐off 1.19. Tau deposition was measured as standardized uptake value ratios (SUVR) in medial‐temporal lobe (MTL) and neocortical (NEO) regions. Linear mixed effect models correcting for age, sex, and years of education were used to investigate whether Aβ‐status (dichotomous) and MTL and NEO tau deposition (continuous, normalized SUVR values) were associated with change over time on the MTB‐composite.

**Result:**

N=100 CU participants (age 70.5±7.8, 63% female, 16.8±2.5 years of education, 27% Aβ+) completed MTB baseline assessments. Of those, n=91 had at least one follow‐up assessment available (7.9±2.4 months after baseline) and n=64 had a second follow‐up assessment (14.6±3 months after baseline). Compared to Aβ‐ individuals, the Aβ+ group showed greater decline in MTB‐composite scores (Time*Aβ+ status: *β* = ‐0.43, 95%CI[‐0.72 – ‐0.14], *p*=.005, Figure 1). Greater MTL and NEO tau deposition were also associated with greater decline in MTB‐composite scores (Time*MTL tau: *β* = ‐0.30, 95%CI[‐0.48 – ‐0.13], *p*=.001, Time*NEO tau: *β* = ‐0.20, 95%CI[‐0.38, ‐0.01], *p*=.042, Figure 2).

**Conclusion:**

These initial findings suggest that the MTB can capture AD‐related cognitive decline and thereby may provide a promising tool to remotely monitor cognition in preclinical AD research and clinical trials.